# Thermoreversible Cross-Linking of Furan-Containing Ethylene/Vinyl Acetate Rubber with Bismaleimide

**DOI:** 10.3390/polym9030081

**Published:** 2017-02-25

**Authors:** Lorenzo Massimo Polgar, Erik Hagting, Wouter-Jan Koek, Francesco Picchioni, Martin van Duin

**Affiliations:** 1 University of Groningen, Department of Chemical Engineering, Nijenborgh 4, 9747 AG Groningen, The Netherlands; l.m.polgar@rug.nl (L.M.P.); erikhagting@hotmail.com (E.H.); wouterjanhk@gmail.com (W.-J.K); martin.vanduin@arlanxeo.com (M.v.D.); 2 Dutch Polymer Institute (DPI), P.O. Box 902, 5600 AX Eindhoven, The Netherlands; 3 ARLANXEO Netherlands, Keltan R&D, Research & Development, P.O. Box 1130, 6160 BC Geleen, The Netherlands

**Keywords:** rubber, thermal reversibility, cross-linking, copolymerization, post-polymerization modification, ethylene/vinyl acetate

## Abstract

A proof of principle for the use of Diels–Alder (DA) chemistry as a thermoreversible cross-linking tool for ethylene–vinyl acetate (EVA) rubber is demonstrated using two differently prepared amorphous furan-functionalized EVA rubbers. The first is an EVFM terpolymer of ethylene, vinyl acetate, and furfuryl methacrylate. The second is an EVA-*g*-furan product, resulting from the reaction of maleated EVA with furfurylamine. Both furan-containing EVA rubbers have been cross-linked with bismaleimide (BM) via a DA coupling reaction to yield final products with similar cross-link density. The BM cross-linked EVFM terpolymer products display rubber properties similar to the ones of peroxide-cured EVA rubbers with similar cross-link densities, whereas the rubber properties of the BM cross-linked EVA-*g*-furan correspond to those of a rubber with a higher cross-link density. The preparation of the EVA-*g*-furan was up-scaled to a small internal mixer, which also allowed compounding with carbon black and mineral oil in the same step. Compounding with carbon black results in reinforcement of the EVA rubber (i.e., enhanced strength), and does not interfere with the reprocessing via the retro DA reaction.

## 1. Introduction

Ethylene/vinyl acetate copolymers (EVA) can be tuned from crystalline plastics to amorphous rubbers by adjusting the vinyl acetate (VA) content. EVAs with 40%–80% VA are soft and flexible materials with little to no crystallinity, which yield elastic products upon cross-linking. Their excellent resistance against heat, weathering, ozone, UV, and oil combined with good processability makes EVA rubbers highly suitable for automotive applications with stringent high-temperature requirements, such as gaskets, seals, and hoses [1,2,3,4]. EVA rubbers have also found common use as photovoltaic insulators [5,6,7,8], cable insulants [1,3,4,9,10,11,12,13], hot melt adhesives [2,3,4,10], coatings [2,10], emulsion paints [2], and shoe soles [3,4,14].

The superior environmental resistance of EVA rubber is the result of the absence of unsaturated C=C bonds in the polymer backbone [1]. Unfortunately, the absence of any unsaturation prevents the use of commonly applied sulfur vulcanization for EVA rubber [9,15]. The main methods for EVA cross-linking are peroxide-curing, high-energy irradiation, and siloxane/moisture curing [2,3,4,9,16]. These cross-linking methods are associated with side-reactions, like chain scission and degradation that compete with the cross-linking reaction, and the formation of irregular networks [15,17,18,19]. Moreover, these curing methods are not thermally reversible, making recycling of waste EVA rubber products impossible.

**Scheme 1:**
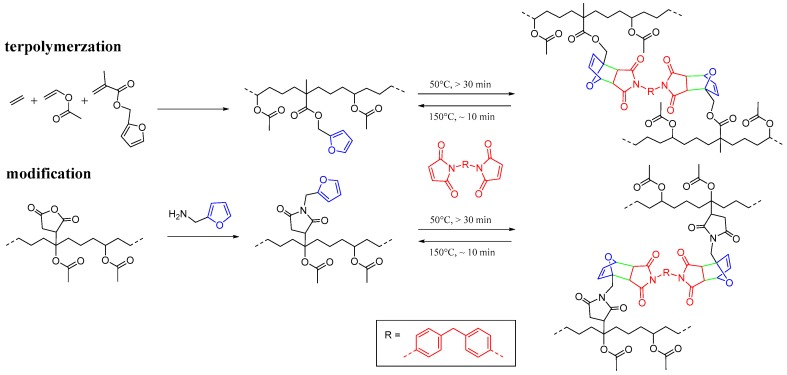
Furan-functionalized ethylene–vinyl acetate (EVA) via terpolymerization (**top**) and post-polymerization modification (**bottom**) and subsequent thermoreversible cross-linking with bismaleimide via (retro) Diels–Alder (DA) chemistry.

Societal trends towards limitation of plastic and rubber waste and the need for more sustainable industrial materials make reversible cross-linking of rubber an interesting technology [19]. Its broad applicability [20,21,22,23], relatively fast kinetics, mild reaction conditions [20,24], and low coupling and high decoupling temperature render the (retro) Diels–Alder (DA) reaction quite suitable for reversible cross-linking of polymers [21,22,25,26,27]. In our previous work, thermoreversible cross-linking of ethylenepropylene (EPM) rubbers was successfully performed using (retro) DA furan-maleimide cross-linking [28]. To implement similar reversible furan-maleimide cross-linking to EVA rubber, a certain degree of functionalization has to be introduced along the saturated EVA polymer backbone—for example, via copolymerization and post-polymerization modification [29]. High-pressure terpolymerization of ethylene, VA, and a furan-containing monomer in a solution process can be used for this purpose. Another approach is post-polymerization modification of EVA rubber via peroxide-initiated, free-radical grafting [21,30,31,32,33,34,35]. Nowadays, the maleation is a mature process and EVA polymers with different degrees of maleic anhydride (MA) are commercially available. Unfortunately, free-radical grafting of MA is often accompanied by a certain amount of undesired side-reactions such as chain-scission and irreversible cross-linking reactions [31,36,37]. Nevertheless, both methods will be pursued to introduce a furan functionality on the EVA rubber backbone (Scheme 1). For both furan-containing EVA rubbers, the addition of an electron-poor bismaleimide (BM) should enable thermoreversible DA cross-linking [22,27,38,39,40]. The VA content of both EVA products is chosen to be approximately 70 wt %, because the effects of cross-linking on material properties are more pronounced as the starting material is fully amorphous.

The first goal of this work is to study the effect of the way the functional furan groups are introduced on the EVA polymer chain (terpolymerization versus post-polymerization modification) on the properties of thermoreversibly cross-linked EVA rubbers. The reprocessability and material performance of the BM cross-linked EVA rubbers will also be compared with peroxide-cured EVA. The furan-functionalization of the maleated EVA and the subsequent DA cross-linking was up-scaled from a solution process to an internal mixer. EVA gum rubbers are usually compounded with reinforcing fillers, such as carbon black [41,42,43], and oil to improve the strength while maintaining good processing. Carbon black also acts as a stabilizer, retarding rubber degradation by oxidation and preventing discoloration [44]. The furan-functionalized EVA is therefore also compounded with carbon black and oil to investigate whether carbon black affects the thermoreversible DA cross-linking and, in addition, still reinforces thermoreversibly cross-linked EVA.

## 2. Materials and Methods 

### 2.1. Materials

The non-modified ethylene/vinyl acetate (VA) copolymer rubber (EVA rubber, Levamelt 700, 30 wt % ethylene, *M*_n_ = 35 kg/mol, polydispersity index (PDI) = 9) and the maleated EVA copolymer rubber (EVA-*g*-MA, 29 wt % ethylene, 69 wt % VA, 1.41 wt % MA, *M*_n_ = 31 kg/mol, PDI = 17) were kindly provided by ARLANXEO, Dormagen, Germany. The ethylene/VA/furfuryl methacrylate (FM) terpolymer (EVFM, 38 wt % ethylene, 60 wt % VA, 2.1 wt % FM, *M*_n_ = 46 kg/mol, PDI = 2.7) was synthesized from ethylene, VA, and FM in *t*-butanol as solvent and using 2,2-azobis 2,4-dimethyl valeronitrile (ADVN) as initiator. The EVA-*g*-MA precursor was dried under vacuum at 175 °C for 1 h to convert any diacid present into cyclic anhydride [28,45]. Carbon black N550 was kindly provided by Teijin Aramid. Octadecyl-1-(3,5-di-*tert*-butyl-4-hydroxy phenyl) propionate (99%), 1,1-(methylenedi-4,1-phenylene)bismaleimide (BM, 95%), dicumyl peroxide (DCP, 98%), dioctyl sebacate (DOS, ≥97%), tetrahydrofuran (THF, >99.9%), toluene (99.8%), and acetone (>99.5%) were all purchased from Sigma-Aldrich (Zwijndrecht, The Netherlands) and used as received as reversible cross-linker, phenolic anti-oxidant, peroxide cross-linker, lubricant, and solvents, respectively. Furfurylamine (FFA, Sigma-Aldrich, ≥99%) was freshly distillated before use.

### 2.2. Methods

#### 2.2.1. Terpolymerization of Ethylene, Vinyl Acetate, and Furfuryl Methacrylate

Seven-hundred forty-three grams of *t*-butanol, 1488 g VA, 2.0 g FM, and 2.5 g ADVN were added to a 5 L high-pressure autoclave under continuous stirring. After removal of oxygen, 1062 g of ethylene was added and the temperature was raised to 61 °C. A pressure of approximately 380 bar was reached. The pressure was kept at 380 bar during the polymerization by adding ethylene. After 30 min, a solution of 122 g *t*-butanol, 157 g VA, and 28 g FM was continuously added over a period of 8.5 h. The acrylate was added in two steps to avoid homopolymerization and/or long FM sequences in the EVFM polymer. After polymerization for 10 h, the polymer solution was transferred to a blow-down tank and residual ethylene was removed. After the removal of unreacted VA and *t*-butanol under vacuum, 615 g of polymer (EVFM) consisting of 38 wt % ethylene, 60 wt % VA, and 2.1 wt % FM (determined by ^1^H-NMR) was obtained. 

#### 2.2.2. Furan Functionalization of EVA-*g*-MA

EVA-*g*-MA rubber was dissolved in THF to afford a 10 wt % solution to which 3 equiv FFA (with respect to the MA in EVA-*g*-MA) was added. The reaction mixture was left to stir at room temperature for 5 h in a closed system and, subsequently, precipitated into a 7-fold of demineralized water under mechanical stirring. The furan-functionalized polymer (EVA-*g*-furan) was obtained as white threads. The polymer product was dried at 50 °C up to constant weight. Finally, the intermediate amide–acid was compression molded for 15 min at 175 °C and 100 bar to ensure the complete conversion to the cyclic imide.

#### 2.2.3. Diels–Alder Cross-Linking

EVA-*g*-furan or EVFM rubber was dissolved in THF to afford a 10 wt % solution to which 1000 ppm phenolic antioxidant and 0.5 equiv BM (with respect to the furan content in the rubber) were added. When homogeneous mixtures were obtained, the majority of the solvent was evaporated in the fume hood by blowing over air. The residual solvent was removed in a vacuum oven at 50 °C. Sample bars of the obtained brownish mixtures were obtained by preheating the materials in a mold at 140 °C for 5 min and compression molding them at 140 °C and 100 bar for 30 min.

#### 2.2.4. Peroxide Curing of EVA

EVA rubber was mixed with the DCP peroxide in an internal mixer (Brabender Messenkneder, Type W 30 EHT, Duisburg, Germany) at 50 rpm and 50 °C. After homogenizing 26 g of EVA rubber (70% fill factor) for 4 min, the desired amount of DCP (1.5 or 3.0 parts per hundred rubber (phr)) was added, and after another 6 min a homogeneous mixture was obtained. Subsequent vulcanization of the obtained mixtures was performed by preheating the samples in a mold at 160 °C for 5 min and compression molding them at 160 °C and 50 bar for 35 min.

#### 2.2.5. Compounding of EVA-*g*-Furan

Rubber compounds with filler and plasticizer were also prepared in the internal mixer (Table 1). Homogenization of 28 g of EVA-*g*-furan was performed at 50 rpm and 50 °C for 2 min. A preblended mixture of 55 phr of carbon black, 15 phr of DOS, and 1000 ppm phenolic antioxidant was added to the mixer and mixed in for 3 min. Next, 0.5 equiv thermoreversible BM cross-linker (with respect to the furan content of the rubber) or 1.6 phr of the DCP peroxide was added to the mixer, which was operated for an additional 6 min. The products were compression molded at 160 °C and 50 bar for 32 min. Finally, the BM cross-linked samples were thermally annealed in an oven at 50 °C for 3 days.

### 2.3. Characterization

Gel permeation chromatography (GPC) was performed using triple detection (refractive index, viscosity, and light scattering) consisting of a Viscotek Ralls detector (Malvern Instruments Ltd., Malvern, United Kingdom), Viscotek Viscometer Model H502 (Malvern Instruments Ltd., Malvern, United Kingdom), and Shodex RI-71 Refractive Index detector (Showa Denko Europe GmbH, Munich, Germany). The separation was carried out by utilizing a guard column (PL-gel 5 µm Guard, 50 mm) and two columns (PL-gel 5 µm MIXED-C, 300 mm) from Agilent Technologies, Amstelveen, The Netherlands. THF (containing 0.01 M lithium bromide) was used as eluent with a flow rate of 1.0 mL/min. The number- and weight-averaged molecular weight (*M*_n_ and *M*_w_; polydispersity index [PDI] = *M*_w_/*M*_n_) was calculated relative to polymethylmethacrylate standards via refractive index, viscosity, and light scattering. ^1^H-NMR spectra were recorded on a Varian Mercury Plus 400 MHz spectrometer (Varian Medical Systems Inc, Palo Alto, CA, USA) with CDCl_3_ as solvent. Elemental analysis (N, C, and H) of the rubber products was performed on a Euro EA elemental analyzer after washing the samples thoroughly in acetone to remove any unreacted BM. The oxygen content was calculated via the mass balance. The number of functional groups per chain (#/chain) was calculated from the determined nitrogen content and the molecular weight. The cross-link density [XLD] was determined from swelling experiments in toluene. The swelling test was performed by weighing a rubber sample (approximately 500 mg) in a 20 mL vial and immersing it in 15 mL toluene until equilibrium swelling was reached (3 days). The sample was weighed after removing the toluene on the surface with a tissue (W1) and was then dried under vacuum at 110 °C until a constant weight was reached (W2). The cross-link density was calculated using the Flory–Rehner Equation (1) with *V*_R_ as the volume fraction of rubber in the swollen sample (2), *V*_S_ the molar volume of the solvent (toluene, 0.1063 L/mol at room temperature), χ the interaction parameter (0.133 for toluene–EVA with 70 mol % VA [13,46]), and ρ the density (930 g/mL for EVA and 870 g/mL for toluene) [47].

[XLD] = (ln(1 − *V*_R_) + *V*_R_ + χ*V*_R_^2^)/(2*V*_S_(0.5*V*_R_ − *V*_R_^1/3^)) (1)

*V*_R_ = *W*_2_/(*W*_2_ + (*W*_1_ − *W*_2_) × (ρ_EVM-g-furan_/ρ_toluene_)) (2)

Differential scanning calorimetry (DSC) was performed using a TA Instruments Q1000 (TA Instruments, New Castle, DE, USA) under a nitrogen atmosphere. The sample for DSC was weighed (10–17 mg) in an aluminum pan, which was then sealed. The glass transition temperature (*T*_g_) was determined by the inflection point method using Universal Analysis software provided by TA Instruments. The rubber products were analyzed in heating/cooling cycles from −60 to 200 °C using heating and cooling rates of 10 °C/min. Four cycles were applied for each sample. Tensile test specimens were prepared by compression molding 500 mg of product into 45 mm long, 5 mm wide, and 1 mm thick rectangular bars. Compression molding was performed on a Taunus Ton Technik V8UP150A press (Friederiksdorf, Germany), equipped with a temperature controller. The tensile tests were performed on an Instron 5565 (Instron, High Wycombe, United Kingdom) with a clamp length of 15 mm, according to the ASTM D4-112 standard. A strain rate of 500 ± 50 mm/min was applied. For each measurement, 10 samples were tested and the 2 outliers with the highest and the lowest values were excluded. The data presented are averages of the other 8 tests. The figures show the median stress–strain curve. Hardness Shore A was measured using a Bareiss Durometer (Heinrich Bareiss Prüfgerätebau GmbH, Oberdischingen, Germany), according to the ASTM D2240 standard. Test samples with a thickness of 2.0 ± 0.1 mm were prepared by compression molding. Average values were obtained from 10 measurements. Taking into account the viscoelastic behavior of EVA rubber, the load reading was taken 50 s after placing the force-loaded indenter on the rubber surface. Compression set tests were performed according to the ASTM D931 standard using a homemade device and cylindrical samples with a thickness of 6.0 ± 0.1 mm and a diameter of 13.0 ± 0.1 mm, which were prepared by compression molding. The samples were compressed to 75% of their original thickness for 70 h at room temperature and relaxed for 30 min at 50 °C.

## 3. Results

### 3.1. Characteristics of (Cross-Linked) EVA Gum Rubber

EVFM and EVA-*g*-furan and their BM cross-linked products were thoroughly extracted and dried before characterizing them by elemental analysis, GPC, DSC, and swelling tests (Table 2). The amount of nitrogen in EVA-*g*-furan is representative for the degree of conversion of the grafted maleic anhydride into the succinimide. Likewise, the cross-linking conversion of both EVA-*g*-furan and EVFM was determined from the molar nitrogen content of the extracted vulcanizates. These results were used to calculate the number of functional and cross-linked groups on the rubber chain as a measure of cross-link density. 

The elemental analysis results confirm the composition of the EVFM terpolymer as determined by ^1^H-NMR (identical numbers obtained for the VA and FM content of 38 wt % and 2.1 wt %, respectively). The findings for the furan-functionalization of EVA-*g*-MA with FFA (>95%) are in line with results previously reported for the facile and complete furan-functionalization of EPM-*g*-MA [28]. The *M*_n_ and PDI do not change upon furan-functionalization of EVM-*g*-MA. For the BM cross-linked EVFM and EVA-*g*-furan, the amount of BM still connected to the rubber after extraction points at a relatively high degree of conversion of the furan functionality (95% and 91% for the BM cross-linked EVFM and EVA-*g*-furan, respectively). These conversions correspond to cross-link densities of 9.4 × 10^−5^ and 1.0 × 10^−4^ mol/mL for the BM cross-linked EVFM and EVA-*g*-furan, respectively, which are very similar to those determined from swell tests (8.6 × 10^−5^ and 8.8 × 10^−5^ mol/mL, respectively). The cross-link densities of BM cross-linked EVFM and EVA-*g*-furan are also quite similar, allowing for a fair comparison of the material properties of these cross-linked products. The same holds for the cross-link densities of the reprocessed BM cross-linked EVA rubbers, viz. of 8.4 × 10^−5^ and 8.3 × 10^−5^ mol/mL for EVFM and EVA-*g*-furan, respectively. Finally, it must be noted that the cross-link density of the 3 phr DCP-cured EVA reference is relatively high with respect to the 1.5 phr DCP-cured sample, which may be because the latter has just passed the gel point. The *T*_g_ of the different EVA rubbers is not affected by cross-linking (differences in *T*_g_ values before and after cross-linking are smaller than 1 °C). The difference of 10 °C in *T*_g_ between EVFM and EVA is probably related to the higher VA content in the latter (30 wt % VA vs 38 wt % for EVFM and EVA, respectively).

**Figure 1:**
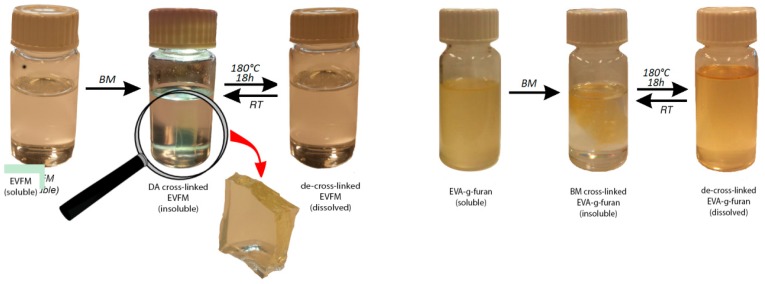
Solubility in 1,2,4-trichlorobenzene at room temperature of non-cross-linked, BM cross-linked, and de-cross-linked samples of EVFM (**left**) and EVA-*g*-furan (**right**).

**Figure 2:**
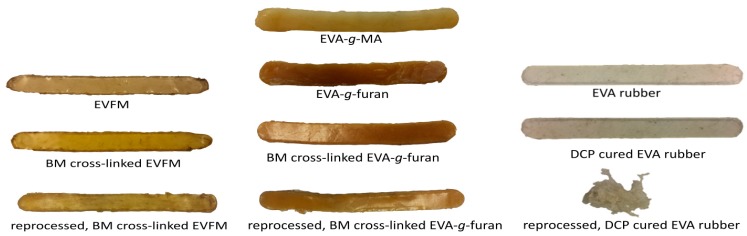
Sample bars of rubber as such and BM/peroxide cross-linked and reprocessed rubber samples of EVFM (**left**); EVA-*g*-furan (**middle**); and EVA rubber (**right**).

While EVA is soluble in most common organic solvents [48], its cross-linked products are expected to be non-soluble and to swell to a certain extent, depending on the degree of cross-linking [49]. Solubility tests in 1,2,4-trichlorobenzene show that both EVA-*g*-furan and EVFM indeed dissolve at room temperature, whereas their corresponding BM cross-linked products do not (Figure 1). Gel contents of the cross-linked products were found to be close to 100% in toluene and 1,2,4-trichlorobenzene. This indicates clearly the formation of a cross-linked network. Heating the 1,2,4-trichlorobenzene to 180 °C results in full dissolution of the BM cross-linked products, which demonstrates that de-cross-linking via the retro-DA reaction takes place at such high temperatures. Finally, the rubber products dissolved at 180 °C were recovered by removing the solvent under vacuum and then shown to be once more insoluble at room temperature, proving that re-cross-linking has taken place. The same conclusion is obtained from the reprocessability of the BM cross-linked sample bars of both materials. Unlike irreversibly peroxide-cured EVA rubber samples, both the BM cross-linked EVFM and EVA-*g*-furan samples can be cut in small pieces and subsequently be compression molded into new coherent sample bars with similar material properties (Figure 2).

### 3.2. Properties of (Cross-Linked) EVA Gum Rubber

**Figure 3:**
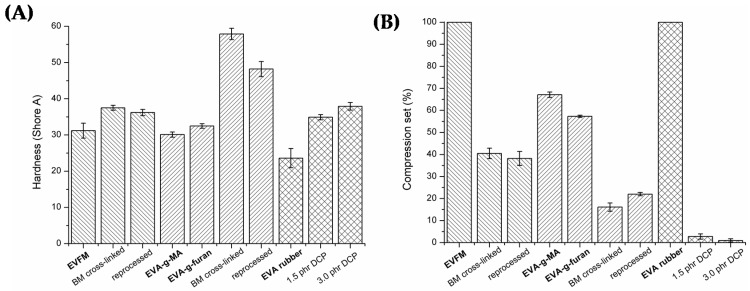
(**A**) Hardness and (**B**) compression set at room temperature of EVFM, EVA-*g*-MA, EVA-*g*-furan, and EVA rubber precursors and their BM/DCP cross-linked and reprocessed products. The error bars indicate ± 1 standard deviation.

The hardness of the different EVA rubber products varies over a very wide range (Figure 3A). EVFM and EVA-*g*-furan initially display a similar hardness, which is significantly higher than that of the non-functionalized EVA rubber. The characteristic increase in hardness that occurs upon cross-linking [50,51] is observed for both BM cross-linked products. The hardness of the BM cross-linked EVFM increases to the same level as that of the DCP-cured EVA rubber reference. The hardness of EVA-*g*-furan, however, increases to a higher level upon BM cross-linking. This may be related to the presence of some irreversible cross-links that are known to form during the peroxide-initiated maleation of the EVA rubber [31,36,37]. Polymer radicals, resulting from hydrogen abstraction by the peroxide-derived alkoxy radicals, may combine, yielding a cross-link instead of reacting with an MA monomer [15,52]. Although this extent of peroxide cross-linking in the EVA-*g*-MA and EVA-*g*-furan products may not be enough to result in significant gel contents in the swell tests, it may still contribute to an increased hardness of the cross-linked polymer. Reprocessing the BM cross-linked samples results in fair retention of the hardness of 97% for EVFM and 83% for EVA-*g*-furan. 

The compression set (CS) at room temperature assesses the ability of a sample to recover its original shape after deformation and, thus, provides a practical measure for the elasticity of a cross-linked rubber. A low CS represents high elasticity and a high CS represents low elasticity. As expected, CS of the starting, non-cross-linked EVFM and EVA products are close to 100%, indicating the absence of any elasticity (Figure 3B). The non-cross-linked EVA-*g*-MA and EVA-*g*-furan products already display a certain degree of elasticity with CS values around 60%, which again may be related to the presence of some irreversible cross-links formed during the peroxide-initiated maleation of the EVA. For apolar polymers grafted with polar groups, like maleated EPM, the presence of phase-separated, polar domains that are rich in (furan-modified) MA grafts, which act as physical cross-links at room temperature, has been shown. However, as demonstrated in another study, such polar clusters do not occur in polar EVA [53]. As expected, BM cross-linking of both EVFM and EVA-*g*-furan results in a decrease of CS with approximately the same step. Upon reprocessing of the BM cross-linked samples, the retention of CS is significantly higher for EVFM than for EVA-*g*-furan (106% versus 73%). Finally, both DCP-cured reference samples show very low CS values close to zero, indicating almost ideal elasticity. In this respect, the relatively high CS values of the BM cross-linked samples with similar cross-link densities may result from the thermoreversible character of the DA cross-links, which allows for their rearrangement under prolonged compression and results in creep or relaxation of the material. 

**Figure 4:**
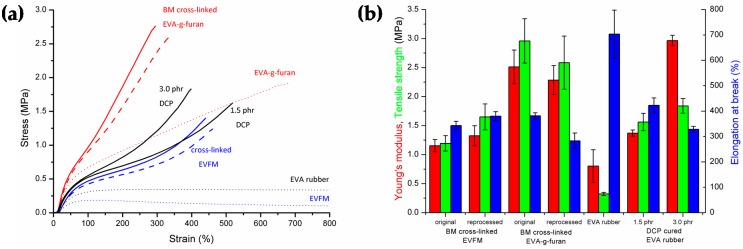
(**a**) Median stress–strain curves of non-cross-linked (dashed lines), BM/peroxide cross-linked (bold lines), and reprocessed (normal lines) EVA-*g*-furan, EVFM, and EVA rubber samples and (**b**) the average values for the Young’s modulus, tensile strength, and elongation.

Tensile tests were performed on all (cross-linked) EVA samples (Figure 4). The non-cross-linked samples are typical gums with low Young’s moduli and tensile strength and extremely high elongation at break. The non-cross-linked EVA-*g*-furan precursor, however, has a significantly higher Young’s modulus and tensile strength and a lower elongation at break than the non-cross-linked EVFM and EVA rubbers. The lower elongation at break may result from the difference in molecular weight (35 kg/mol for EVA-*g*-furan versus 45 kg/mol for EVFM) as larger polymers are more entangled [49,54]. This difference may also be a consequence of the presence of the low amount of irreversible peroxide cross-linking that is known to occur upon the maleation of EVA. All three EVA rubbers show a typical increase in Young’s modulus and tensile strength and a decrease in elongation at break upon cross-linking with respect to their non-cross-linked precursors [46,55,56]. The tensile properties of both thermoreversibly cross-linked EVA rubbers appear to be superior (larger tensile strength at a comparable elongation at break) to those of DCP-cured EVA rubbers with comparable cross-link densities. The Young’s modulus and tensile strength of the BM cross-linked EVA-*g*-furan are also higher than that of the BM cross-linked EVFM, despite their similar cross-link density. Meanwhile, the relative increase in Young’s modulus and tensile strength and decrease in elongation at break with respect to their non-cross-linked precursors is larger for EVFM. This may, again, be due to the aforementioned presence of irreversible cross-links in EVA-*g*-furan as these cross-links would be unable to dissociate. This is in line with the lower retention of these properties upon reprocessing of the BM cross-linked EVA-*g*-furan with respect to the BM cross-linked EVFM. Nevertheless, both materials show high retention of their tensile properties upon reprocessing (~90%). 

### 3.3. Cross-Linked EVA Rubber Compounds

**Figure 5:**
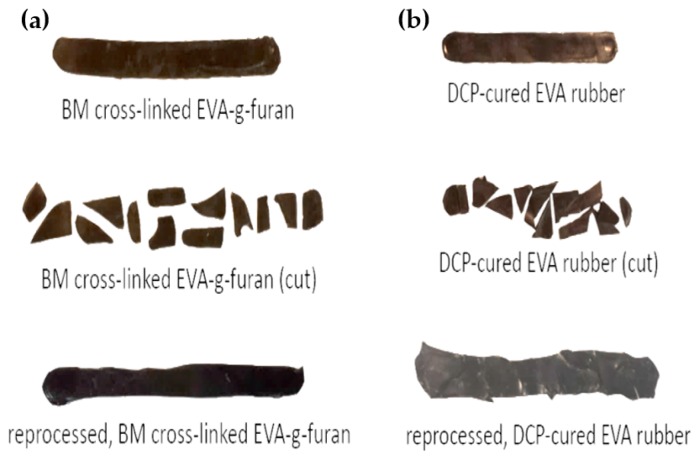
Sample bars of cross-linked, cut, and reprocessed samples of (**a**) BM cross-linked EVA-*g*-furan compounds and (**b**) DCP-cured EVA rubber compounds.

The furan-functionalization and DA cross-linking of EVA-*g*-MA was up-scaled from a solution route to an internal mixer, following a procedure as applied earlier for a maleated EPM [57]. This also allows for first compounding with carbon black and oil, followed by the addition of the BM cross-linker in a one-step process. A common EVA compound formulation [1] was used and modified to replace the DCP cross-linker by 0.5 molar equivalent of BM with respect to the furan content of EVA-*g*-furan, assuming full conversion of the anhydride of EVM-*g*-MA during the preceding melt modification step. The compounding of EVA-*g*-furan with carbon black and oil does not interfere with the DA cross-linking, as gel contents of 100% are obtained from swell tests. The cross-link densities of the EVA compounds were determined in the same way, as the EVA gum rubbers cannot be used in an absolute fashion, because of the presence of the carbon black and oil. Still, the apparent cross-link density does allow for a qualitative comparison. The apparent cross-link densities of both the BM cross-linked EVM-*g*-furan compound and the DCP-cured EVA reference compound (3.1 × 10^−4^ ± 2.4 × 10^−2^, and 2.7 × 10^−4^ ± 3.5 × 10^−2^ mol/mL, respectively) are quite similar, approximately 3 times higher than that of their corresponding gum rubbers. These increased apparent cross-link densities are the result of the presence of carbon black, which is known to adsorb rubber molecules onto its surface and to include them in internal voids. This results in partial immobilization of the rubber chains, restricting the rubber chain mobility and the vulcanizate swelling [58]. Compounding the BM cross-linked EVA-*g*-furan with carbon black does not affect its thermoreversible character, as sample bars remain reprocessable upon compression molding, whereas the DCP-cured reference compounds do not (Figure 5).

**Figure 6:**
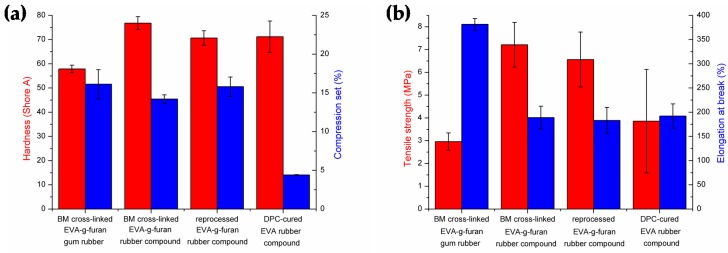
(**a**) Hardness and compression set at room temperature and (**b**) tensile strength and elongation at break of BM cross-linked EVA-*g*-furan gum rubber and compounds, reprocessed compounds, and DCP-cured EVA rubber compounds. The error bars indicate ± 1 standard deviation.

Compounding of the BM cross-linked EVA-*g*-furan with carbon black and mineral oil results in an increase of the hardness and the tensile strength and a decrease of the elongation at break, compared to the BM cross-linked EVA-*g*-furan gum rubber analogues (Figure 6). These effects are a result of the interaction between the rubber and the active carbon black filler. The decrease in elongation at break may also result from the reduced rubber content of the compound upon the introduction of a non-elastic filler [58]. CS as a measure for elasticity is not that much affected by the compounding. The retention of the material properties of the reprocessed rubber compounds is higher than that of the reprocessed gum rubbers. This may be because reinforced compounds have a higher starting level of properties resulting in a relatively lower deterioration of properties upon reprocessing.

The BM cross-linked EVA-*g*-furan compound and the DCP-cured EVA compound have a similar hardness, tensile strength and elongation at break within the error margins. This demonstrates that the cross-links formed as a result of the DA cycloaddition of the grafted furan groups and the BM cross-linker are indeed strong, covalent bonds. Despite their similar cross-link density, however, the BM cross-linked EVA-*g*-furan compound has a significantly higher CS than the DCP-cured samples. This may be due to the thermoreversible character of the DA cross-links, allowing for the rearrangement of the cross-links under prolonged compression. This effect appears to be relatively slow as it is not observed for the relatively high-speed tensile properties. 

## 4. Conclusions

Amorphous EVA rubbers were thermoreversibly cross-linked via DA chemistry using new experimental approaches. First, the furan functionality required for the DA cross-linking reaction was achieved through terpolymerization of ethylene, vinyl acetate, and furfuryl methacrylate, resulting in EVFM, and secondly via post-polymerization modification of a maleated EVA resulting in EVA-*g*-furan. Both products were successfully cross-linked with the bismaleimide cross-linker and shown to be reprocessable in the melt with good retention of their material properties (approximately 90%).

The BM cross-linked EVA-*g*-furan rubbers have superior rubber properties—such as an increased hardness, Young’s modulus, and tensile strength and a lower elongation at break and compression set—than the BM cross-linked EVFM rubbers with a similar cross-link density. This can be explained by the formation of a certain degree of irreversible peroxide cross-links that takes place during peroxide-initiated MA grafting of EVA rubber. Finally, the EVA-*g*-MA rubber was also functionalized with furan, compounded with carbon black and oil and mixed with the BM cross-linker in an internal mixer, which is a far more practical approach than the original solvent route. Compounding with carbon black does not interfere with the reversible DA cross-linking, and the vulcanizate retains its rubber properties upon reprocessing.

## Figures and Tables

**Table 1:** Formulation for the preparation of thermoreversibly cross-linked EVA-*g*-furan rubber compounds.

Component	phr	Time of Addition (min)
EVA-*g*-furan	100	0
Carbon black (N550)	55	2
Lubricant (DOS)	15	2
Antioxidant	0.1	2
BM/DCP cross-linker	2.6/1.6	5

DOS: dioctyl sebacate; BM: bismaleimide; DCP: dicumyl peroxide; phr: parts per hundred rubber.

**Table 2:** Reaction conversion according to elemental analysis.

	*M*_n_ (kg/mol)	PDI (-)	N, C, H → O Content (wt %)	#/Chain (-)	*T*_g_ (°C)	[XLD] (mol/mL)
EVA rubber	35	9.1	<0.01, 64.41, 9.19 → 26.40	–	−11.9	-
EVFM	46	2.7	<0.01, 67.41, 9.87 → 22.72	5.8	−21.7	-
EVA-*g*-MA	31	17	<0.01, 65.40, 9.61 → 24.99	4.4	−12.7	-
EVA-*g*-furan	31	17	0.20, 66.37, 9.52 → 23.91	4.3	−11.0	-
BM cross-linked EVFM	-	-	0.35, 67.34, 9.62 → 22.69	5.5	−22.3	8.62 × 10^−5^ ± 2.0 × 10^−5^
BM cross-linked EVA-*g*-furan	-	-	0.42, 65.16, 9.34 → 25.08	3.9	−9.7	8.75 × 10^−5^ ± 3.0 × 10^−6^
1.5 phr DCP cured EVA	-	-	<0.01, 64.44, 9.04 → 26.52	-	−12.3	4.8 × 10^−5^ ± 1.1 × 10^−5^
3.0 phr DCP cured EVA	-	-	<0.01, 62.47, 8.91 → 28.62	-	−12.7	2.4 × 10^−4^ ± 2.3 × 10^−5^

EVFM: ethylene/VA/furfuryl methacrylate; MA: maleic anhydride; PDI: polydispersity index; [XLD]: cross-link density; *T*_g_: glass transition temperature.
